# Methylation Drivers and Prognostic Implications in Sinonasal Poorly Differentiated Carcinomas

**DOI:** 10.3390/cancers13195030

**Published:** 2021-10-08

**Authors:** Laura Libera, Giorgia Ottini, Nora Sahnane, Fabiana Pettenon, Mario Turri-Zanoni, Alessia Lambertoni, Anna Maria Chiaravalli, Federico Leone, Paolo Battaglia, Paolo Castelnuovo, Silvia Uccella, Daniela Furlan, Carla Facco, Fausto Sessa

**Affiliations:** 1 Unit of Pathology, Department of Medicine and Surgery, ASST Sette-Laghi, University of Insubria, 21100 Varese, Italy; laura.libera@uninsubria.it (L.L.); giorgia.ottini@asst-settelaghi.it (G.O.); pettenonfabiana@gmail.com (F.P.); annamaria.chiaravalli@asst-settelaghi.it (A.M.C.); silvia.uccella@uninsubria.it (S.U.); Daniela.Furlan@uninsubria.it (D.F.); carla.facco@asst-settelaghi.it (C.F.); fausto.sessa@uninsubria.it (F.S.); 2 Division of Otorhinolaryngology, Department of Biotechnology and Life Sciences, ASST Sette-Laghi, University of Insubria, 21100 Varese, Italy; mario.turrizanoni@asst-settelaghi.it (M.T.-Z.); alessia.lambertoni@gmail.com (A.L.); federico.leone88@gmail.com (F.L.); paolo.battaglia@uninsubria.it (P.B.); paolo.castelnuovo@asst-settelaghi.it (P.C.)

**Keywords:** CpG island methylator phenotype, *SMARCB1*, INI1, *IDH2*, sinonasal neuroendocrine cancer, undifferentiated cancer, LINE-1 methylation

## Abstract

**Simple Summary:**

Poorly differentiated sinonasal carcinomas (PDSNCs) are rare neoplasms that include a wide spectrum of malignancies characterized by alteration in different epigenetic mechanisms (SWI/SNF complex, *IDH2*, *NUT*). The aim of our study was to verify if the identification of specific genetic and epigenetic alterations can be useful to recognize different clinico-pathological subsets of PDSNCs to guide treatment decisions. In our cohort, 14 cases showed alterations in SWI/SNF complex or *IDH2* genes, which were associated with a higher global DNA methylation level and worst prognosis. The integration of genetic and epigenetic features appears to be a good strategy to improve the clinico-pathological classification of these tumors and to recognize distinct prognostic entities that deserve tailored clinical management.

**Abstract:**

Background: Poorly differentiated sinonasal carcinomas (PDSNCs) are rare and aggressive malignancies, which include squamous cell carcinoma (SCC), sinonasal undifferentiated carcinoma (SNUC), and neuroendocrine carcinomas (NEC). Several epigenetic markers have been suggested to support the histopathological classification, predict prognosis, and guide therapeutic decision. Indeed, molecularly distinct subtypes of sinonasal carcinomas, including SMARCB1-INI1 or SMARCA4 deficient sinonasal carcinoma, isocitrate dehydrogenase (IDH)-mutant SNUC, ARID1A mutant PDSNCs, and NUT carcinomas, have recently been proposed as separate entities. Identification of aberrant DNA methylation levels associated with these specific epigenetic driver genes could be useful for prognostic and therapeutic purpose. Methods: Histopathological review and immunohistochemical study was performed on 53 PDSNCs. Molecular analysis included mutational profile by NGS, Sanger sequencing, and MLPA analyses, and global DNA methylation profile using LINE-1 bisulfite-PCR and pyrosequencing analysis. Results: Nine SWI/SNF complex defective cases and five *IDH2* p.Arg172x cases were identified. A significant correlation between INI-1 or *IDH2* defects and LINE-1 hypermethylation was observed (*p* = 0.002 and *p* = 0.032, respectively), which were associated with a worse prognosis (*p* = 0.007). Conclusions: Genetic and epigenetic characterization of PDSNCs should be performed to identify distinct prognostic entities, which deserved a tailored clinical treatment.

## 1. Introduction

Sinonasal carcinomas are rare neoplasms, representing approximately 5% of all head and neck tumors [1]. A significant proportion of them are high-grade epithelial neoplasms, which can be collectively designated poorly differentiated sinonasal carcinomas (PDSNCs). PDSNCs encompass a heterogeneous spectrum of highly aggressive malignancies, including squamous cell carcinoma (SCC), sinonasal undifferentiated carcinoma (SNUC), NUT carcinomas, and neuroendocrine carcinomas (NEC). Although these entities have well-known histogenetic and biological diversities, they frequently share an overlapping morphology, whereby histopathology alone is frequently not sufficient to render a precise and clinically meaningful diagnosis [2,3].

Several immunohistochemical markers have been suggested to support the histopathological classification, and recent advances in molecular profiling have led to the identification of molecular markers that can be relevant to predict prognosis and for therapeutic decision-making [4,5,6]. Particularly, molecular abnormalities of members of the switch/sucrose non-fermentable (SWI/SNF) complex, which is involved in chromatin regulation and gene expression processes, have been related to subsets of PDSNCs with specific clinico-pathological features [7]. The SWI/SNF complex has been shown to be highly mutated across diverse cancer types displaying undifferentiated, anaplastic, or rhabdoid phenotypes [8]. Indeed, molecularly distinct subtypes of sinonasal carcinomas, including SMARCB1-INI1 or SMARCA4 deficient sinonasal carcinoma, isocitrate dehydrogenase (IDH)-mutant SNUC, ARID1A mutant PDSNCs, and NUT carcinomas (harboring NUT-variant fusions), have recently been proposed as separate entities [2,9,10,11].

SMARCB1 (INI-1)-deficient sinonasal carcinoma has been identified in about 100 cases so far in the world, showing a poor prognosis, with a mean survival of 22 months [12,13]. Most of these cases had previous diagnoses of SNUC, but also of other PDSNCs subtypes, including non-keratinizing squamous cell carcinoma and, very rarely, plasmacytoid and rhabdoid morphologies [14,15,16,17]. SMARCA4-deficient sinonasal carcinoma is a very infrequent tumor, previously rendered as a SNUC, with very few cases reported so far [9].

*IDH* mutant sinonasal cancer is a recently discovered molecular subtype with fewer than 50 described cases so far, which have been identified among SNUC and large cells NEC (LCNEC) subgroups [18,19].

In line with data reported in other tumor sites, Dogan S et al. showed that *IDH* somatic mutations induce a hypermethylator phenotype and define a distinct molecular and prognostic subgroup of sinonasal carcinomas [20,21].

Finally, ARID1A mutations have been reported in five *IDH* wild-type PDSNCs), whereas *NUT* gene rearrangements, leading to differentiation arrest through chromatin deregulation, are specific molecular markers of NUT carcinomas [21,22,23,24].

Interestingly, all the genes mentioned above play a role in multiple processes, regulating the epigenetic status of the cell through chromatin remodeling, DNA methylation, and histone protein methylation. Moreover, these genes show mutually exclusive alterations in PDSNCs, suggesting that these aberrations must have interchangeable effects on tumorigenesis in this site, and that an epigenetic deregulation could be the hallmark of these cancers.

In this work, we have collected a large and well-characterized series of 53 PDSNCs in order to verify if (i) the main epigenetic genes involved in these tumors can be putative DNA methylation drivers leading to a tumor methylator phenotype and (ii) aberrant DNA methylation levels associated with specific driver gene alterations can be useful to recognize different clinico-pathological subsets of PDSNCs to guide treatment decisions.

## 2. Materials and Methods

### 2.1. Case Series Presentation

The study was performed on a retrospective series of 53 consecutive cases of PDSNCs (Table 1, Table S1).

All these patients were treated in a single tertiary-care referral university hospital between January 2008 and December 2018. Clinical, surgical, and follow-up data were retrospectively retrieved from the institutional database for skull base cancers.

To evaluate the site, origin, and extent of the neoplasm, all patients underwent a preoperative clinical evaluation through nasal endoscopy and radiological studies, including computed tomography (TC) and contrast-enhanced magnetic resonance imaging (MRI). Neck ultrasound, total body contrast-enhanced CT scan, and/or PET scan were performed in all cases to rule out systemic dissemination of disease. In order to define the histological diagnosis and plan the most adequate treatment, an endoscopic incisional biopsy was performed in each case. All cases were discussed and managed by a head and neck multidisciplinary team, including otorhinolaryngologist, neurosurgeon, medical oncologist, radiotherapist, pathologist, radiologist, and anesthesiologist in order to select the most appropriate set of multimodal therapies for each patient, combining surgery, neoadjuvant, and adjuvant treatments according to current protocols [25].

All patients included in this series were surgically treated using endoscopic assisted approaches tailored to the extension of disease and ranging from an exclusive endonasal resection (EER) to an expanded resection (ERTC, endoscopic resection with transnasal craniectomy). In selected cases of locally advanced cancer, a combined endoscopic and transcranial resection was performed (CER). Neoadjuvant chemotherapy was performed in suitable cases, according to histology driven protocols previously described, which included a “TPF” regimen (docetaxel, cisplatin, and 5-fluorouracil) for SCC and SNUC and a “PE-AI” regimen (cisplatin/etoposide, and Adriamycin/ifosfamide) for cancers displaying neuroendocrine features. The number of cycles ranged according to response and toxicity [25].

Post-operative radiotherapy was delivered in locally advanced malignant tumours (pT3–pT4) using the intensity modulated technique (IMRT) or heavy-ion therapy (proton beam or carbon-ion therapy). Elective neck irradiation was considered in selected high-risk cases [25]. In case of positive surgical margins, a platinum-based adjuvant chemotherapy was delivered concomitant to radiotherapy. All patients were followed up in accordance with a specific protocol that included endoscopic nasal examination and contrast-enhanced MRI of the head at scheduled intervals [25]. Detailed information was collected about patient’s professional and extraprofessional risk factors, as suggested by Franchi et al., such as smoking habits, complete occupational history, leisure activities, and environmental or domestic exposures [26]. All procedures were carried out in accordance with the Helsinki Declaration, and the study was approved by the local ethics committee.

### 2.2. Histopathological Review and Immunohistochemical Study

Histopathological slides were reviewed by two head and neck pathologists and an endocrine pathologist. Diagnoses of SNUC and SCC were rendered according to the World Health Organization classification of tumors, 4th edition [11]. In brief, a diagnosis of sinonasal undifferentiated carcinoma was made only in totally undifferentiated epithelial tumors without any morphologic evidence of glandular or squamous differentiation, negative NUT, and absent of focal and weak immunopositivity of neuroendocrine markers (chromogranin A and synaptophysin); a diagnosis of squamocellular carcinoma was made morphologically and then by subclassifying the keratinizing tumors from non-keratinizing. NECs were classified according to the recently proposed common classification framework for neuroendocrine neoplasms of different anatomical locations proposed by IARC/WHO [27]. In detail, the diagnosis of NEC was rendered on the basis of the observation of a clear-cut poorly differentiated neuroendocrine morphology and of high proliferation indexes (mitosis and Ki67 proliferation index), along with the immunohistochemical expression of cytokeratins and at least two general neuroendocrine markers among synaptophysin, chromogranin A, and INSM1. The absence of p63 and p40 positivity and NUT negativity was requested for diagnosis. NECs were subclassified in small cell and large cell subtypes. Cases in which a coexistence of NEC and non-neuroendocrine carcinoma was observed were designed as mixed neuroendocrine-non neuroendocrine neoplasms (MiNEN), and the different components were detailed in qualitative and quantitative terms [28]. Immunohistochemistry for NUT, INI1/SMARCB1, BRG1/SMARCA4, SMARCA2 ARID1A, p53, and RB was performed in all cases.

The immunohistochemical analysis was performed on formalin-fixed, paraffin-embedded tumor sections collected on Superfrost Plus slides. Tumor sections were processed automatically on BenchMark ULTRA instrument (Ventana) with OptiView DAB detection kit or Ultraview DAB detection kit (Ventana) using the antibodies listed in Table S2.

### 2.3. Targeted Next Generation Sequencing (NGS) Analysis

Tumor DNA was extracted from three representative 8 µm-thick sections obtained from 47 FFPE samples available for the molecular analyses and neoplastic areas were manually microdissected in order to have at least 50% of tumor cells. DNA was extracted using Maxwell^®^ DNA FFPE Kit and Maxwell 16 system (Promega, Madison, WI, USA) according to the manufacturer’s protocol. Each sample was quantified using Qubit dsDNA High Sensitivity Assay kit (Invitrogen, Thermo Fisher Scientific Inc., Waltham, MA, USA).

A gene-targeted NGS analysis was performed on a subset of 30 DNA samples using the Human Actionable Solid Tumor Mutations QIAseq DNA Panel (DHS-101Z, Qiagen, Hilden, Germany) that analyzes 22 oncogenes (*BRAF, PDGFRA, EGFR, KRAS, NRAS, KIT, AKT1, ALK, CTNNB1, ERBB3, ESR1, FOXL2, GNA11, GNAQ, IDH1, IDH2, MET, RAF1, RET, ERBB2, PIK3CA,* and *TP53*). A targeted amplicon-based library was constructed as described in a previous work of our group according to the manufacturer protocol [29]. Barcoded libraries were pooled together at 8pM and sequenced on an Ion S5 XL System (A27214, Thermo Fisher Scientific) using Ion 530 chip (Thermo Fisher Scientific). Unmapped BAM (uBAM) files were imported into CLC Genomics Workbench (Qiagen Bioinformatics, Germany, version 12) and mapped on the human hg19 genome. Sequencing data were analyzed using the Biomedical Genomics Analysis plugin and filtered ensuring a coverage of at least 100X and a variant allele frequency (VAF) higher than 5%.

### 2.4. Sanger Sequencing Analysis of IDH2 Exon 4

The sequence of *IDH2* exon 4 was amplified at the annealing temperature of 55 °C using the GoTaq^®^ G2 Flexi DNA Polymerase (Promega, Madison, WI, USA) and the following primers: forward primer 5′-TGTCCTCACAGAGTTCAAGCT-3′ and reverse primer 5′-GATCCCCTCTCCACCCTG-3′. Sequencing was performed on purified PCR products by using BigDye^®^ Terminator v.1.1 Cycle Sequencing kit (Thermo Fisher Scientific Inc, Waltham, MA, USA) and run on SeqStudio^®^ Genetic Analyzer (Thermo Fisher Scientific Inc., Waltham, MA, USA) after purification with DyeEx 2.0 Spin Kit^®^ (Qiagen, Hilden, Germany). Sequences were analyzed by visual inspection using SeqA^®^ software v.7 (Thermo Fisher Scientific Inc., Waltham, MA, USA) by two independent molecular biologists.

### 2.5. Multiplex Ligation-Dependent Probe Amplification Assay of SMARCB1

Deletions/duplications analysis of *SMARCB1* gene was performed using SALSA MLPA (multiplex ligation-dependent probe amplification) probemix P258 (MRC-Holland, Amsterdam, The Netherlands) according to the manufacturer’s protocol. Electrophoresis of the amplified products was performed with a SeqStudio Genetic Analyzer and the electropherograms were checked with GeneMapper Software version 6 (Thermo Fisher Scientific). Output data were analyzed comparing the samples with three healthy controls using Coffalyser.net MLPA analysis software (MRC-Holland). The cut-off values used to evaluate gene/exon imbalances were 0.8 and 1.2 for loss and gain of signal, respectively.

### 2.6. LINE-1 Methylation Analysis

The methylation status of global LINE-1 (GenBank accession number M80343.1) was evaluated by bisulfite-PCR and pyrosequencing. Bisulfite modification of genomic DNA (300 ng) was performed with EZ DNA Methylation Kit (Zymo Research, Irvine, CA, USA) according to the manufacturer’s recommendations. Bisulfite-modified DNA was amplified and sequenced addressing four CpG sites by using LINE-1 primers and protocol previously reported by Stefanoli et al. [30]. Human methylated and non-methylated (WGA) DNA sets (Zymo Research, Irvine, CA, USA) were used as positive and negative controls in each experiment.

### 2.7. Statistical Analysis

Statistical analysis was performed using Student’s t-test. Survival curves were calculated using the Kaplan–Meier estimator test. Multivariate analysis was performed using Cox regression analysis for those variables that resulted significant with Kaplan–Meier estimator test. A *p* value of <0.05 was considered significant. The GraphPad v.5.0 (GraphPad Software Inc., San Diego, CA, USA) and MedCalc 11.2.0.0 (MedCalc Software, Ostend, Belgium) software were used for statistical analyses.

## 3. Results

### 3.1. Clinico-Histopathological Results and Immunohistochemical Study

Histological and clinicopathological features of 53 PDSNCs, namely 33 SCC, 14 NEC, and 6 SNUC, are reported in Table 1 and, in more details, in Table S1.

The average age of onset in the whole series was 61.5 years old, and no significant differences were observed between the three histological subtypes (mean age: SNUC 63 years; NEC 61.5 years; SCC 60.9 years). In our series, 21 patients reported a professional exposure, and 24 patients were cigarette smokers. Most of these patients developed a keratinizing squamocellular carcinoma (KSCC) (15 and 13 cases, respectively). The stage at diagnosis was prevalently T3–T4 (49/53 cases), N0 (50/53 cases), and M0 (52/53 cases), and the most frequent site of origin was the ethmoidal sinus (36/53 cases).

**Figure 1:**
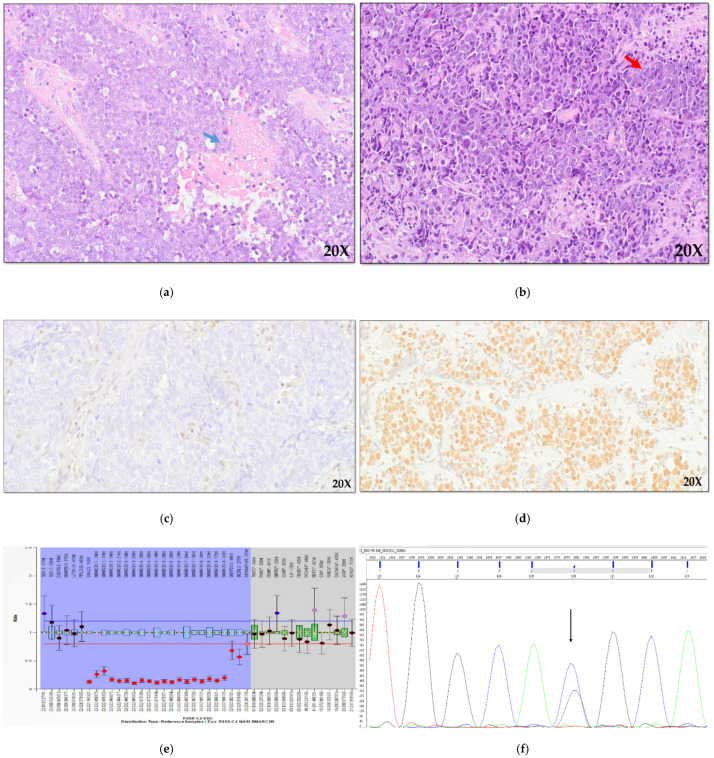
Example of two representative poorly differentiated sinonasal carcinomas. On the left side, an NKSCC ((**a**), haematoxylin-eosin stain, x20) with ribbon-like growth pattern, absent maturation and necrotic areas (blue arrows), showing total loss of SMARCB1 protein in the tumor area except for the normal stromal cells (**c**) and biallelic loss of *SMARCB1* gene ((**e**), red dots). On the right side, a LCNEC (**b**) composed by medium-large cells arranged in organoid nests (red arrow), showing the presence of SMARCB1/INI1 protein (**d**) with intense nuclear positivity (brown stain) and *IDH2* p.Arg172Thr mutation ((**f**), black arrow).

As regards the histological subtypes, the 33 SCCs comprised 26 KSCCs and 7 non-keratinizing carcinomas (NKSCC, Figure 1a). The 14 NEC cases included seven large cells neuroendocrine carcinoma (LCNEC, Figure 1b), four small cell NEC (SCNEC), and three mixed neuroendocrine-non neuroendocrine neoplasms (MiNEN). In detail, the MiNEN subgroup comprehend one case of LCNEC with a NKSCC component, one SCNEC with an intestinal-type adenocarcinoma (ITAC) area, and one LCNEC+ITAC. The remaining six cases were diagnosed as SNUC.

Immunohistochemical analysis of SMARCB1 (INI1), SMARCA4 (BRG1), and SMARCA2 (BRM) revealed loss of these proteins in four cases NKSCC (Figure 1a), in four cases of KSCC and in one case of MiNEN (LCNEC + NKSCC). In one case of SNUC, we found the loss of only SMARCA4 and SMARCA2 subunits, and finally, in one case of KSCC, the loss of only SMARCA4 protein was observed. Immunohistochemical analysis of ARID1A protein was possible on 48 cases and did not identify any case showing the loss of the protein.

As regards p53 protein, complete protein loss was observed in 10 SCC, 5 NEC, and 2 SNUC and aberrant nuclear accumulation in 9 NEC, 4 SCC and 3 SNUC; no abnormal pattern was present in 18 SCC and 1 SNUC. Nuclear expression of Retinoblastoma protein was lost in 17 SCC, 6 NEC, and in only 1 SNUC; in four cases, the immunohistochemical reaction was not evaluable.

### 3.2. Targeted NGS Analysis

NGS analysis by Human Actionable Solid Tumor Mutations QIAseq DNA Panel was possible on a subset of 30 samples for which a good DNA quality was available. This analysis showed good coverage with a mean read depth of 725X (585X minimum coverage and 977X maximum coverage) and identified a total of 1389 variants. These variants were filtered out when the depth of coverage was less than 200X, VAF was lower than 5%, and they were annotated as synonymous or listed in 1000 Genome Project.

Thus, a total of 26 variants with a deleterious effect on protein functions were detected in 16 out of 30 sinonasal carcinomas analyzed (seven cases showed more than one mutation), of which 24 were missense mutations, 1 was a non-sense mutation, and 1 was an in-frame insertion. Among these variants, 21 were annotated as likely pathogenic or pathogenic (class 4/5), and 5 were reported as variants with uncertain significance (VUS, class 3). As reported in Table S3, the most involved genes were *PIK3CA* (six pathogenic variants and four VUS), *TP53* (seven pathogenic variants), and *IDH2* (five pathogenic variants).

**Figure 2:**
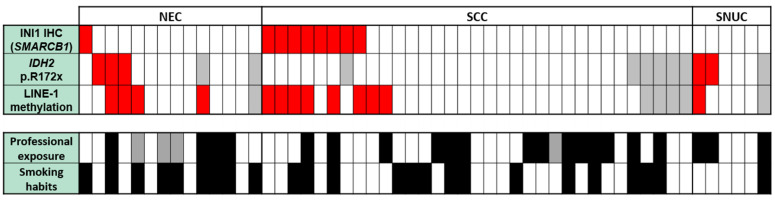
Schematic overview of molecular and clinical characterization of 53 poorly differentiated epithelial sinonasal tumors. Red cell: SMARCB1 loss or presence of IDH2 mutation or LINE-1 hypermethylation (>70%); black cell: presence of professional exposure/smoking habits; grey cell: data not available.

Considering the histological subtypes, six out of nine NEC (67%), 8 out of 18 SCC (55%), and two out of three SNUC (67%) showed at least one pathogenic variant. Comparing NEC and SCC cases, we observed a higher number of mutations in NEC, especially in *TP53* and *IDH2* genes (Figure S1). As all *IDH2* gene mutations occurred in codon 172 (p.Arg172Gly p.Arg172Thr and p.Arg172Ser), and in the light of recent literature, we extended the analysis of this region to the whole series using hot-spot direct sequencing (Figure 1b), but no additional *IDH2*-mutated cases were found (Table S1, Figure 2) [31].

### 3.3. SMARCB1 Loss Analysis

*SMARCB1* copy number analysis was possible in 41 out of 53 sinonasal carcinomas with a good quality DNA, including 8 INI1-negative cases and 33 cases with a normal expression of INI1 protein.

**Figure 3:**
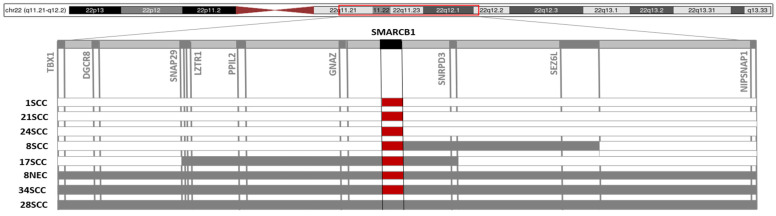
*SMARCB1* analysis by MS-MLPA. All cases INI1-negative (1SCC, 21SCC, 24SCC, 8SCC, 17SCC, 8NEC, 34SCC, and 28SCC, enlisted on the left of the figure) showed monoallelic (grey) or biallelic (red) loss of *SMARCB1* locus, which, in five cases, was extended to a larger region of chromosome 22q (monoallelic loss of adjacent genes, in grey).

All the INI1-negative cases showed *SMARCB1* deletions (Figure 3): homozygous deletions were identified in seven cases, including five SCC and two NEC. Only one heterozygous *SMARCB1* deletion was identified, in case 28SCC (Figure 3), suggesting that in this neoplasm a mutation in the second allele might have occurred. Interestingly, a heterozygous deletion extending to contiguous genes were found in five cases, as shown in details in Figure 3, but, due to the small number of cases, we were not able to assess if this could have a clinical meaning.

### 3.4. SMARCB1 Loss and IDH2 Mutations Correlate with Global DNA Hypermethylation

As mutated forms of *SMARCB1* and *IDH2* have been demonstrated to be involved in the epigenetic dysregulation, we aimed to check the levels of global DNA methylation in the tumors showing SMARCB1 loss or *IDH2* mutations.

Quantitative LINE-1 methylation analysis was possible in 47 out of 53 sinonasal carcinomas and in five normal nasal tissues for comparison. In normal tissues, LINE-1 methylation rate ranged from 45% to 60% (average 52.8 ± 2.5%), while in PDSNCs, the distribution of LINE-1 levels varied from 22.2% to 77.4% (average 57.5 ± 2.5%).

**Figure 4:**
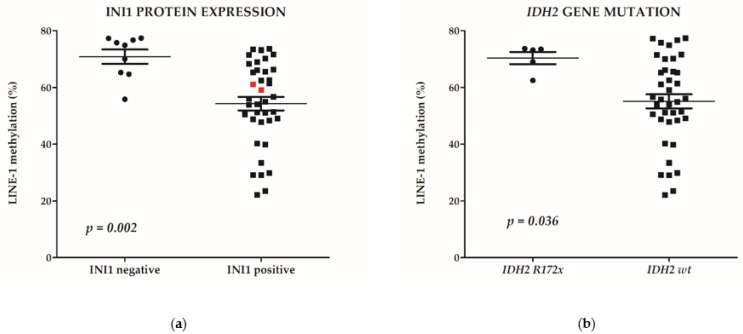
Both INI1 immunonegativity (*p* = 0.002) and *IDH2* p.Arg172x mutation (*p* = 0.036) correlate to LINE-1 hypermethylation. LINE-1 methylation level (%) is reported in *y*-axis as a continuous variable. (**a**) LINE-1 methylation levels of INI1 negative versus INI1 positive cases. Red dots are representative of two cases showing BRG1 loss of immunoreactivity; (**b**) LINE-1 methylation levels of IDH2 mutant versus *IDH2 wild-type* (*wt*) cases.

A total of 13 PDSNCs showed LINE-1 methylation percentages higher than 70%, and this value corresponded to the LINE-1 methylation level detaching the fourth quartile of the data set. These 13 tumors were classified as LINE-1 hypermethylated cases and included six of the nine (67%) INI1-negative tumors, three of the five (60%) *IDH2* p.Arg172x (or R172x) mutated, and four PDSNCs without any specific molecular or immunohistochemical alterations (Figure 2). INI1 negative cases exhibited significantly higher methylation levels with respect to INI1-positive samples (mean value of 70.9% versus 54.3%, respectively; *p* = 0.002, Figure 4a). Analogously, *IDH2* p.Arg172x mutated cases displayed higher LINE-1 methylation levels (70.4%) with respect to *IDH2 wild-type* cases (55.1%, *p* = 0.036, Figure 4b).

LINE-1 methylation levels were also correlated with all the clinico-pathological, immunohistochemical, and genetic features considered in this study, but no other association was found.

### 3.5. PDSNCs with Epigenetic Alterations Show a Worse Prognosis

**Figure 5:**
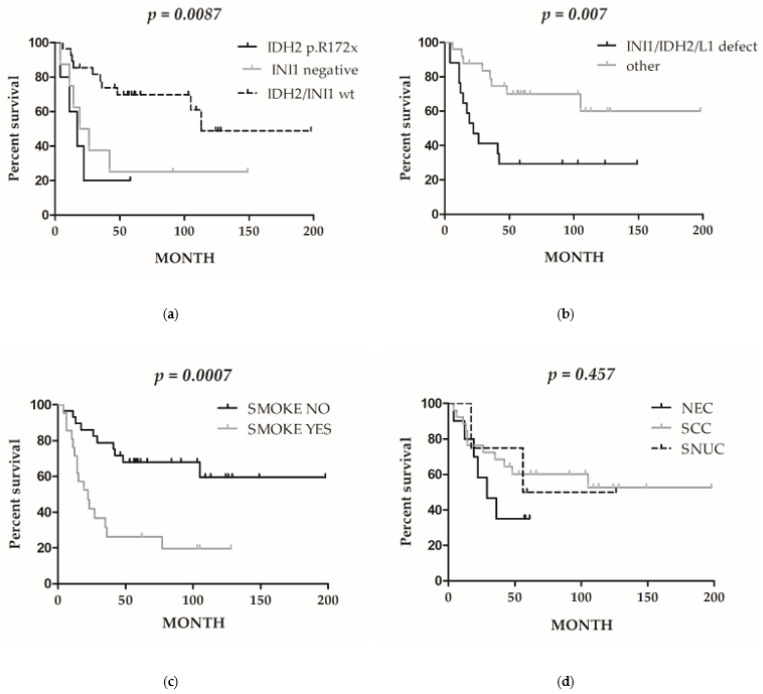
Univariable survival analyses according to molecular markers (**a**,**b**), smoking habits (**c**), and histotype (**d**). (**a**) Outcome (disease-specific survival) of *IDH2*-mutated (*n* = 5), INI1 negative (*n* = 9), and *IDH2*/INI1 wild-type (*n* = 37) cases; (**b**) Outcome of *IDH2*-mutated, INI-1 negative or LINE-1 hypermethylated cases combined together (presence of at least one variable, *n* = 27) versus wild-type or LINE-1 < 70% cases (*n* = 24); (**c**) outcome of smokers (*n* = 22) versus non-smokers (*n* = 29); (**d**) disease-specific survival analysis comparing the three histotypes: NEC (neuroendocrine carcinomas), SCC (squamocellular carcinoma), and SNUC (sinonasal undifferentiated carcinoma).

Disease-specific survival analysis (DSS, Table 1) was performed on 51 out of 53 patients. Considering each molecular marker as a single parameter, we observed a significant worse survival rate in patients harboring LINE-1 hypermethylation (*p* = 0.02) and *IDH2* p.Arg172x mutation (*p* = 0.02) (Figure S2). A trend towards statistical significance was observed for INI1-negative cases (*p* = 0.09, Figure S2). Interestingly, by combining the three variables together and considering the presence of at least one of them as a defective subgroup, the DSS analysis showed a better stratification, as the INI1/*IDH2*/LINE-1 defective subset had 60% versus 29% of survival rates at a 150 month follow-up time (*p* = 0.007, Figure 5a).

Among clinicopathological features, smoking habits identified a subgroup of patients with a worse DSS compared to non-smokers, indicating a reduced survival rate in tobacco users (*p* = 0.0007, Figure 5b), whereas histotype and professional exposure had no prognostic meaning in this series (Figure 5d, Figure S2).

Interestingly, among the four variables that were significant at survival analysis (LINE-1, INI1 defect, *IDH2* p.Arg172x, and smoking habits), INI1 defect, *IDH2* p.Arg172x mutation, and smoking habits were independent prognostic factors, as resulted from the Multivariable Cox regression (Table 2), whereas LINE-1 hypermethylation was excluded by the multivariable regression model (*p* > 0.1), because this variable was associated with INI1 and *IDH2* mutations (Figure 4, Table 2).

## 4. Discussion

Recent evidence shows that somatic mutations in several driver genes are intrinsically connected with DNA methylation patterns in cancer, and that the mutation-methylation relationships described in many tumors could potentially be used to classify malignancies [32].

Sinonasal cavities are anatomical areas from which a wide histological diversity of neoplasms emerges. Among epithelial neoplasms of these sites, a significant subset shows morphological high-grade features and aggressive clinical behavior and may be collectively designed poorly differentiated sinonasal carcinomas (PDSNCs). Despite these morphological similarities, PDSNCs encompass several histogenetically and biologically heterogeneous neoplasms. For this reason, a diagnosis primarily based on histological features is challenging for the pathologist, and an integrated analysis of biological and morphological features is mandatory to recognize distinct prognostic entities that deserve tailored clinical management [10,33].

Recent studies of molecular profiling of PDSNCs have demonstrated frequent alterations of chromatin modulators, i.e., SWI/SNF subunits (SMARCB1, SMARCA2, SMARCA4, and ARID1A) or of proteins leading to CpG island methylator phenotype (CIMP) by inhibiting the TET-demethylation pathway (i.e., *IDH2*) [34]. Additionally, *NUT* gene rearrangements, leading to differentiation arrest through chromatin deregulation, are specific molecular markers of NUT carcinomas. Altogether, these genetic alterations confirm the crucial role of key epigenetic players in the tumorigenesis of PDSNCs, suggesting that specific interconnections between tumor genomes and epigenomes deserve to be investigated in these tumors [32].

The working hypothesis of this study was that the main epigenetic genes involved in PDSNCs can be putative DNA methylation drivers, leading to aberrant DNA methylation levels in specific tumor subsets. To address this issue, for the first time with this work, a quantitative methylation analysis of LINE-1 sequences (long interspersed nuclear elements, which accounts for 17% of the whole genome) was integrated with the study of all the immunohistochemical and molecular markers currently considered useful to distinguish the novel clinico-pathological entities in this site.

Although we know that our study is limited by the small sample size, due to the rarity of these tumors, we could, nevertheless, analyze a well-characterized cohort of 53 PDSNCs, including NEC, SNUC, and SCC patients treated with CRT or surgery +/− CRT. Survival analysis from our cohort is congruent with previous reports of low survival rates [35]. The immunohistochemical and molecular study demonstrated the main involvement of SMARC family members and of *IDH2* gene in our series, while no immunohistochemical anomalies were detected for ARID1A and NUT proteins. Globally, loss of SWI/SNF subunits (SMARCB1, SMARCA4, SMARCA2) was observed in 11 PDSNCs. As expected, these genes showed mutually exclusive alterations in all but one case, which was a SNUC exhibiting simultaneous loss of SMARCA4 and SMARCA2 proteins. SMARCB1 loss was detected in most of these cases (9/11), including eight SCCs and one mixed neuroendocrine/non-neuroendocrine neoplasm (MiNEN), composed of a LCNEC and a NKSCC. These results suggest that deregulation of the SWI/SNF nucleosome remodeling complex, through one of its many components, is a critical step in disease progression of high-grade SCC. To date, SMARCB1 loss has been described in about 100 sinonasal carcinomas worldwide, mostly among SNUC, and a worse outcome has been demonstrated in these cases, while more rarely, this alteration was observed in NKSCC and in tumors with plasmacytoid and rhabdoid morphologies [14,15,16,17]. Although, in our series, none of the six SNUC showed *SMARCB1* loss, we could confirm low survival rates in patients with SMARCB1-deficient carcinomas, suggesting that, regardless of the histological features observed in PDSNCs, this marker identifies a specific biological entity with a potential impact for prognosis and targeted therapeutic options [36].

As regards *IDH2* gene, 5 of 53 (9.4%) PDSNCs showed a pathogenic mutation in the hotspot codon p.Arg172, and this variant was always mutually exclusive with loss of SWI/SNF subunits. *IDH2* mutant tumors comprised three NECs and two SNUCs. From a clinical point of view, *IDH2*-mutant cases showed an aggressive clinical behavior that was remarkably different from that observed in cases without these mutations. This finding appears to be in contrast with recently published data by Riobello C. et al. and Gloss S. et al. [31,37]. However, IDH-mutant sinonasal cancer is a recently discovered molecular subtype with about a hundred described cases so far, and further future efforts are needed to better classify the *IDH2*-mutated subset in this site. Interestingly, in line with published data in other malignancies, three recent epigenetic studies of sinonasal tumors reported that *IDH* somatic mutations induce a CpG island methylator phenotype, reminiscent of *IDH*- mutant gliomas where pathways linking IDH to tumorigenesis have been described for the first time [20,21,38,39,40]. Beyond diagnostic considerations, these findings have significant implications for therapy with IDH inhibitors, which have been recently approved to treat acute myeloid leukemia [41]. In our study, we found that *IDH2* mutation was associated with higher LINE-1 methylation levels in PDSNCs, confirming that, as a global DNA methylation assay, LINE-1 analysis may be a promising marker to quickly assess a methylator phenotype in these tumors. Similarly, OhKa F et al. proposed LINE-1 methylation assay as a good global DNA methylation surrogate to identify Glioma-CpG Island methylator phenotype (G-CIMP) in *IDH*-mutant glioma [42].

A key finding of our work was that a larger subset of PDSNCs (13/47; 28%) exhibited global LINE-1 hypermethylation, and that this marker was associated with a significant worse survival rate. Moreover, LINE-1 hypermethylation appeared to be significantly associated not only with *IDH2* mutations but also with INI1 loss. Interestingly, the combination of the three variables in DSS analysis strongly improved the prognostic stratification of the patients, showing that the presence of at least one marker (INI-1 loss and/or *IDH2* mutation and/or LINE hypermethylation) was greatly associated with a poor DSS.

As expected, the multivariable Cox regression analysis showed that only INI1 defect, *IDH2* mutation, and smoking habits were independent prognostic factors. Of note, smoking habits, which are well-known risk factors for this site and have a strong negative prognostic value in our cohort, were not associated with the DNA hypermethylation or hypomethylation profiles [26]. In other anatomic sites, i.e., lung cancer, tobacco smoking was associated with LINE-1 hypomethylation, *TP53* mutation, and high rates of copy number alterations [43]. In light of this consideration, further studies are needed in PDSNCs to better elucidate the interconnections between epigenetic and genetic alterations in a subset of tumors where tobacco smoking is the etiological factor.

Altogether, our results suggest that the combined analysis of global LINE-1 hypermethylation status with INI-1 and *IDH2* alterations allows the recognition of a distinct molecular subset of PDSNCs characterized by an aggressive biologic behavior. The identification of this tumor profile could lead to targeted therapeutic options and improved overall disease-specific survival.

## Figures and Tables

**Table 1:** Clinicopathological features of 53 poorly differentiated epithelial sinonasal tumors.

	Whole Series	SNUC	NEC ^1^	SCC ^2^
**No.**	53	6	14	33
**Age (mean, years)**	61.5	63	61.5	60.9
**Sex**				
Male	35	3	12	20
Female	18	3	2	13
TNM				
T1–T2	4	0	1	3
T3–T4	49	6	13	30
N0	50	6	13	31
N1	0	0	0	0
N2–N3	3	0	1	2
M0	52	6	13	33
M1	1	0	1	0
**Site of origin**				
Ethmoidal sinus	36	5	12	19
Maxillary sinus	13	1	1	11
Frontal sinus	3	0	1	2
Sphenoidal sinus	1	0	0	1
**Professional exposure**				
Yes	21	3	4	14
No	28	3	7	18
Not available	4	0	3	1
**Smoking habits**				
Yes	24	1	9	14
No	29	5	5	19
**Follow-up status**				
Median survival (months)	105	127	29	n.r. ^3^
Died	26	3	8	15
Alive	25	3	5	17
Not available	2	0	1	1

^1^ NEC (neuroendocrine carcinomas): seven large-cell NEC; four small-cell NEC; one mixed NKSCC-LCNEC; two mixed adenocarcinoma (ADC)-NEC (1 ADC-SCNEC; 1 ADC-LCNEC). ^2^ SCC (squamocellular carcinoma): 26 keratinizing SCC; 7 non-keratinizing SCC. ^3^ n.r. (not reached): 60% still alive at 200 months follow-up.

**Table 2:** Results from Cox proportional hazards regression estimation (backward).

Covariate	OR	95% CI of OR	*p*
INI1 negativity	4.11	1.39–12.15	0.0105
*IDH2* p.Arg172x	5.99	1.74–20.69	0.0046
Smoking habits	3.50	1.35–9.07	0.0102
LINE-1 hypermethylation	-	-	n.s.

LINE-1 hypermethylation is excluded by the regression model, as it is not an independent variable (*p* > 0.1); indeed, it is associated with INI1 negativity and *IDH2* p.Arg172x. OR: odd’s ratio; CI: confidence interval; n.s.: not significant.

## Data Availability

The data presented in this study are available in the article or in supplementary material.

## Supplementary Materials

Supplementary data are available online at https://www.mdpi.com/article/10.3390/cancers13195030/s1. Table S1: Comprehensive clinical, histological, immunohistochemical and molecular features of of 53 poorly differentiated epithelial sinonasal tumors. Table S2: Immunohistochemistry primary antibody list. Table S3: NGS results. Figure S1: Distribution of pathogenic variants in SCC vs. NEC. Figure S2: Univariable Survival Analyses according to INI1, *IDH2*, LINE-1 status and professional exposure.
