# Supplementary material for: Methylation Drivers and Prognostic Implications in Sinonasal Poorly Differentiated Carcinomas
Source: Cancers (Basel). 2021 Oct 8;13(19):5030. doi: 10.3390/cancers13195030 (PMC8507885; doi:10.3390/cancers13195030)
Supplement: Supplementary file 1 [file cancers-13-05030-s001.zip › cancers-1399681-SI.pdf]

Table S1. Comprehensive clinical, histological, immunohistochemical and molecular features of of 53 poorly differentiated epithelial sinonasal tumors.

						TNM STAGE																
**ID**	**Age**	**Gender**	**Site of origin**	**-**	**Revised Diagnosis**	**T**	**N**	**M**	**INI1 IHC (SMARCB1)**	**BRG1 IHC (SMARCA4)**	**BRM IHC (SMARCA2)**	**p53 IHC**	**Rb IHC**	**ARID1A IHC**	**LINE1 methylation level (%)**	**SALSA MLPA P258 (SMARCB1)**	***IDH2* exon 4 mutation**	**NGS results**	**Professional exposition**	**Smoking habits**	**DSS (Disease Specific Survival)**	**DSS time (months)**
25SCC	80	f	e	SCC	KSCC	4b	0	0	+	+	+	+	-	+	29.1	wt	wt	wt	1	0	0	53
11SCC	57	f	mx	SCC	KSCC	3	0	0	+	+	+	na	na	+	39.9	wt	wt	na	1	0	1	105
33SCC	69	f	e	SCC	KSCC	3	3b	0	+	+	+	+	+	+	48.9	wt	wt	MUT	0	1	na	na
9SCC	70	f	e	SCC	KSCC	4b	0	0	+	+	+	+++	-	+	51.5	wt	wt	MUT	0	1	1	6
27SCC	46	f	e	SCC	KSCC	3	0	0	+	+	+	+++	+	+	55.0	wt	wt	MUT	0	0	0	46
1SCC	46	f	e	SCC	KSCC	4a	0	0	-	/	/	-	-	+	65.3	BIALLELIC LOSS SMARCB1	wt	na	0	0	0	149
7SCC	57	f	s	SCC	KSCC	4a	0	0	+	+	+	+	+	+	70.2	wt	wt	na	1	0	0	124
19SCC	59	f	mx	SCC	KSCC	2	0	0	+	+	+	+	-	na	na	na	na	na	1	1	1	77
29SCC	86	m	mx	SCC	KSCC	4b	0	0	+	+	+	+	+	+	22.2	wt	wt	wt	1	0	1	13
6SCC	65	m	e	SCC	KSCC	4b	0	0	+	+	+	-	-	+	33.4	wt	wt	MUT	1	1	0	128
30SCC	67	m	mx	SCC	KSCC	4a	0	0	+	+	+	-	-	+	47.9	wt	wt	MUT	1	1	1	14
20SCC	79	m	mx	SCC	KSCC	3	0	0	+	+	+	-	+	+	51.1	wt	wt	MUT	1	0	0	66
18SCC	63	m	mx	SCC	KSCC	4a	0	0	+	+	+	+	-	+	54.1	wt	wt	na	0	1	1	35
4SCC	40	m	e	SCC	KSCC	4b	0	0	+	+	+	+	-	+	65.4	na	wt	na	0	0	0	198
15SCC	45	m	e	SCC	KSCC	4a	0	0	+	+	+	-	+	na	71.7	wt	wt	na	0	0	0	103
21SCC	40	m	mx	SCC	KSCC	4b	0	0	-	/	/	+	-	+	74.9	BIALLELIC LOSS SMARCB1	wt	wt	1	1	1	14
24SCC	70	m	mx	SCC	KSCC	4b	0	0	-	/	/	+	+	+	77.3	BIALLELIC LOSS SMARCB1	wt	wt	0	1	1	4
34SCC	66	m	e	SCC	KSCC	4b	0	0	-	/	/	+	+	+	77.4	BIALLELIC LOSS SMARCB1 MONOALLELIC LOSS 22q	wt	wt	1	1	1	11
16SCC	68	m	e	SCC	KSCC	4a	0	0	+	+	+	-	-	na	na	na	na	na	1	1	1	15
23SCC	68	m	fr	SCC	KSCC	4a	0	0	+	+	+	+++	+	na	na	na	na	na	0	0	0	84
2SCC	68	f	e	SCC	KSCC ***	3	0	0	+	+	+	+	-	+	40.2	wt	wt	wt	na	0	1	48
13SCC	52	m	mx	SCC	KSCC***	4a	0	0	+	+	+	+	-	+	51.3	wt	wt	wt	0	1	0	105
22SCC	55	m	e	SCC	KSCC***	2	0	0	+	+	+	+	+	+	61.4	wt	wt	wt	1	1	0	62
5SCC	64	m	e	SCC	KSCC***	3	0	0	+	+	+	-	-	+	68.4	na	na	na	0	0	0	129
14SCC	68	f	e	SCC	KSCC****	4a	0	0	+	-	+	-	+	+	59.1	wt	wt	MUT	1	1	0	103
26SCC	78	m	mx	SCC	KSCC****	3	0	0	+	+	+	+++	+	+	29.2	wt	wt	na	1	0	0	13
10SCC	65	f	e	SCC	NKSCC	4a	0	0	+	+	+	+	-	+	23.5	na	wt	wt	0	0	1	113
12SCC	70	f	e	SCC	NKSCC	4a	0	0	+	+	+	+	-	+	50.6	wt	wt	na	0	0	0	109

3SCC	42	f	fr	SCC	NKSCC	4b	0	0	-	/	/	-	-	+	64.7	na	na	na	0	0	1	41
28SCC	48	m	mx	SCC	NKSCC	4b	0	0	-	/	/	-	+	+	70.0	MONOAL-LELIC LOSS 22q	wt	MUT	0	0	1	26
8SCC	43	m	e	SCC	NKSCC	2	0	0	-	/	/	+	-	+	75.8	BIALLELIC LOSS SMARCB1	wt	na	0	0	1	42
17SCC	76	m	e	SCC	NKSCC	4b	0	0	-	/	/	+	+	+	76.7	BIALLELIC LOSS SMARCB1 MONOAL-LELIC LOSS OF SNAP29-GNAZ + SNRPD3	wt	wt	0	0	0	91
32SCC	67	m	e	SCC	NKSCC	4b	3b	0	+	+	+	+	+	na	na	na	na	na	0	1	1	6
7NEC	54	f	e	NEC	LCNEC	4b	0	0	+	+	+	+++	+	+	70.0	wt	p.Arg172Gly	MUT	0	0	1	4
6NEC	73	m	e	NEC	LCNEC	4b	0	0	+	+	+	+++	+	+	49.2	wt	wt	MUT	1	1	1	36
12NEC	22	m	e	NEC	LCNEC	2	0	0	+	+	+	+++	+	+	53.9	wt	wt	MUT	0	0	0	61
5NEC	53	m	e	NEC	LCNEC	3	3	0	+	+	+	-	+	+	66.4	na	na	na	0	1	1	10
19NEC	76	m	e	NEC	LCNEC	4b	0	0	+	+	+	+++	-	+	73.2	wt	p.Arg172Thr	MUT	1	1	1	22
16NEC	48	m	mx	NEC	LCNEC	4b	0	0	+	+	+	+++	-	+	73.7	wt	p.Arg172Thr	MUT	0	0	0	58
3NEC	79	m	e	NEC	LCNEC	4b	0	0	+	+	+	+++	-	na	na	na	na	na	1	1	1	27
1NEC	74	m	e	NEC	mixed NEC+ADC *	4b	0	0	+	+	+	+++	na	+	56.7	na	wt	MUT	na	1	0	0
15NEC	63	m	e	NEC	mixed NEC+ADC **	4b	0	0	+	+	+	-	+	+	65.6	wt	wt	na	1	1	0	19
8NEC	72	m	fr	NEC	mixed NEC+NKSCC**	4b	0	0	-	/	/	+++	-	+	55.8	BIALLELIC LOSS SMARCB1 MONOAL-LELIC LOSS 22q	wt	wt	0	1	1	19
9NEC	42	f	e	NEC	SCNEC	4b	0	0	+	+	+	-	-	+	29.9	wt	wt	wt	0	0	1	29
17NEC	73	m	e	NEC	SCNEC	4b	0	0	+	+	+	+++	-	+	62.6	wt	wt	wt	0	0	0	57
18NEC	80	m	e	NEC	SCNEC	4b	0	1	+	+	+	-	na	+	66.1	wt	wt	na	na	1	na	na
4NEC	44	m	e	NEC	SCNEC	4a	0	0	+	+	+	-	+	+	71.5	wt	wt	MUT	nv	1	1	12
2SNUC	74	f	e	SNUC	SNUC	4a	0	0	+	+	+	+	+	+	48.5	wt	wt	na	0	0	0	59
3SNUC	48	f	e	SNUC	SNUC	4b	0	0	+	+	+	+++	+	+	56.1	wt	wt	na	0	0	0	56

4SNUC	77	f	e	SNUC	SNUC	4b	0	0	+	+	+	-	+	+	62.5	wt	p.Arg172Ser	na	1	0	1	17
1SNUC	63	m	e	SNUC	SNUC	4a	0	0	+	-	-	-	-	+	61.1	wt	wt	wt	0	0	0	126
7SNUC	63	m	e	SNUC	SNUC	4b	0	0	+	+	+	+++	na	+	73.5	wt	p.Arg172Ser	MUT	1	0	1	11
6SNUC	53	m	mx	SNUC	SNUC	4a	0	0	+	+	+	-	+	na	na	na	na	na	1	1	1	23

f=female; m=male; e=ethmoidal sinus; mx=maxillary sinus; fr=frontal sinus; s=sphenoidal sinus; SCC=squamocellular carcinoma; NEC=neuroendocrine carcinoma; SNUC=sinonasal undifferentiated carcinoma; NKSCC=non keratinizing SCC; KSCC= keratinizing SCC; LCNEC=large cell NEC; SCNEC=small cell NEC; ADC=adenocarcinomas (intestinal type); na=not available; +=presence; -=absecnce; +++=accumulation; wt=wild-type; MUT=presence of at least 1 mutation; 1=yes/died; 0=no/alive; *SCNEC; **LCNEC; *** basaloid SCC; **** spindle cell SCC.

Table S2. Immunohistochemistry primary antibody list.

Primary Antibody	Clone	Working Solution	Manufacturer
mouse anti-Pan Keratin	AE1-AE3 & PCK26	pure	Roche
mouse anti-Chromogranin A	LK2H10	pure	Roche
mouse anti-CD45	RP2/18	pure	Roche
mouse anti-Ki67	Mib-1	1/100	Dako
rabbit anti-NSE	MRQ-55	pure	Roche
rabbit anti-NUT	C52B1	1/100	Cell Signaling
mouse anti-p63	4A4	pure	Roche
mouse anti-p40	BC28	pure	Roche
rat anti-Serotonin	YC5/45	1/100	Serotec
rabbit anti-Synaptophysin	SP11	pure	Roche
rabbit anti-S100	SP127	pure	Roche
mouse anti-INI1/SMARCB1	MRQ-27	1月-50	Cell Marque
anti-BRG1/SMARCA4	EPR 3912	1/100	Abcam
rabbit anti-SMARCA2	-	1/400	Sigma-Aldrich
mouse anti-p53	D07	pure	Roche
mouse anti-RB	G3-245, BD	1/100	Pharmigen
rabbit anti-ARID1A	-	1/100	Sigma Aldrich

Table S3. NGS results.

ID	Histitype class	NGS	Coverage	PIK3CA	TP53	IDH2	CTNNB1	EGFR	KRAS	KIT	Other genes*	LINE1
6NEC	NEC	**MUT**	586	WT	**p.Arg175His 75%**	WT	WT	**p.Asp717_Asn718ins SerValAsp 40%**	WT	WT	WT	49.2
4NEC	NEC	**MUT**	642	**p.His1047Leu 83%**	**p.Tyr205Cys 82%**	WT	**p.Asp32Tyr 77%**	WT	WT	WT	WT	71.5
7NEC	NEC	**MUT**	803	**p.Glu707Lys 19%**	**p.Cys176Phe 87%**	**p.Arg172Gly 87%**	WT	WT	WT	WT	WT	70.0
16NEC	NEC	**MUT**	780	WT	WT	**p.Arg172Thr 48%**	WT	WT	WT	WT	WT	73.7
19NEC	NEC	**MUT**	706	**p.Glu707Lys 5%**	WT	**p.Arg172Thr 63%**	WT	WT	WT	WT	WT	73.2
12NEC	NEC	**MUT**	653	WT	**p.Arg282Trp 25%**	WT	WT	WT	WT	WT	WT	53.9
8NEC	NEC	WT	690	WT	WT	WT	WT	WT	WT	WT	WT	55.8
17NEC	NEC	WT	691	WT	WT	WT	WT	WT	WT	WT	WT	62.6
9NEC	NEC	WT	759	WT	WT	WT	WT	WT	WT	WT	WT	29.9
30SCC	SCC	**MUT**	803	WT	WT	WT	WT	WT	**p.Gly12Asp 42%**	WT	WT	47.9
33SCC	SCC	**MUT**	938	**p.Asn345Lys 35%**	**p.Arg306* 28%**	WT	WT	WT	WT	**p.Asp910 Tyr 38%**	WT	48.9
27SCC	SCC	**MUT**	809	**p.Glu707Lys 5%**	**p.Ala138Val 55%**	WT	WT	WT	WT	WT	WT	55.0
9SCC	SCC	**MUT**	850	**p.Glu707Lys 11%**	**p.His193Arg 20%**	WT	WT	WT	WT	WT	WT	51.5
14SCC	SCC	**MUT**	655	**p.Arg524Lys 15%**	WT	WT	WT	WT	WT	WT	WT	59.1
20SCC	SCC	**MUT**	619	**p.Arg524Lys 16% p.Glu707Lys 5%**	WT	WT	WT	WT	WT	WT	WT	51.1
6SCC	SCC	**MUT**	710	**p.Glu545Lys 6% p.His1047Tyr 8%**	WT	WT	WT	WT	WT	WT	WT	33.4
28SCC	SCC	**MUT**	737	**p.His1047Tyr 47%**	WT	WT	WT	WT	WT	WT	WT	70.0
29SCC	SCC	WT	602	WT	WT	WT	WT	WT	WT	WT	WT	22.2
21SCC	SCC	WT	620	WT	WT	WT	WT	WT	WT	WT	WT	74.9
17SCC	SCC	WT	635	WT	WT	WT	WT	WT	WT	WT	WT	76.7
25SCC	SCC	WT	646	WT	WT	WT	WT	WT	WT	WT	WT	29.1

22SCC	SCC	WT	653	WT	WT	WT	WT	WT	WT	WT	WT	61.4
13SCC	SCC	WT	693	WT	WT	WT	WT	WT	WT	WT	WT	51.3
24SCC	SCC	WT	776	WT	WT	WT	WT	WT	WT	WT	WT	77.3
2SCC	SCC	WT	777	WT	WT	WT	WT	WT	WT	WT	WT	40.2
34SCC	SCC	WT	811	WT	WT	WT	WT	WT	WT	WT	WT	77.4
10SCC	SCC	WT	977	WT	WT	WT	WT	WT	WT	WT	WT	23.5
7SNUC	SNUC	**MUT**	752	WT	WT	**p.Arg172Ser 27%**	WT	WT	WT	WT	WT	73.5
4SNUC	SNUC	**MUT**	758	WT	WT	**p.Arg172Ser 93%**	WT	WT	WT	WT	WT	62.5
1SNUC	SNUC	WT	618	WT	WT	WT	WT	WT	WT	WT	WT	61.1

* BRAF, PDGFRA, NRAS, AKT1, ALK, ERBB3, ESR1, FOXL2, GNA11, GNAQ, IDH1, MET, RAF1, RET, ERBB2. Orange tables represent Uncertain Significance.

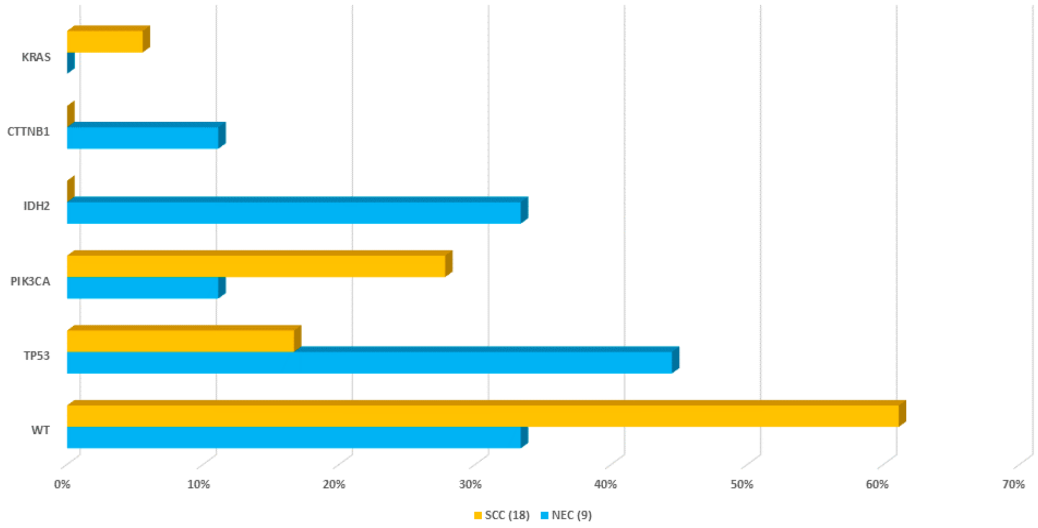

Figure S1. Distribution of pathogenic variants in SCC vs NEC.

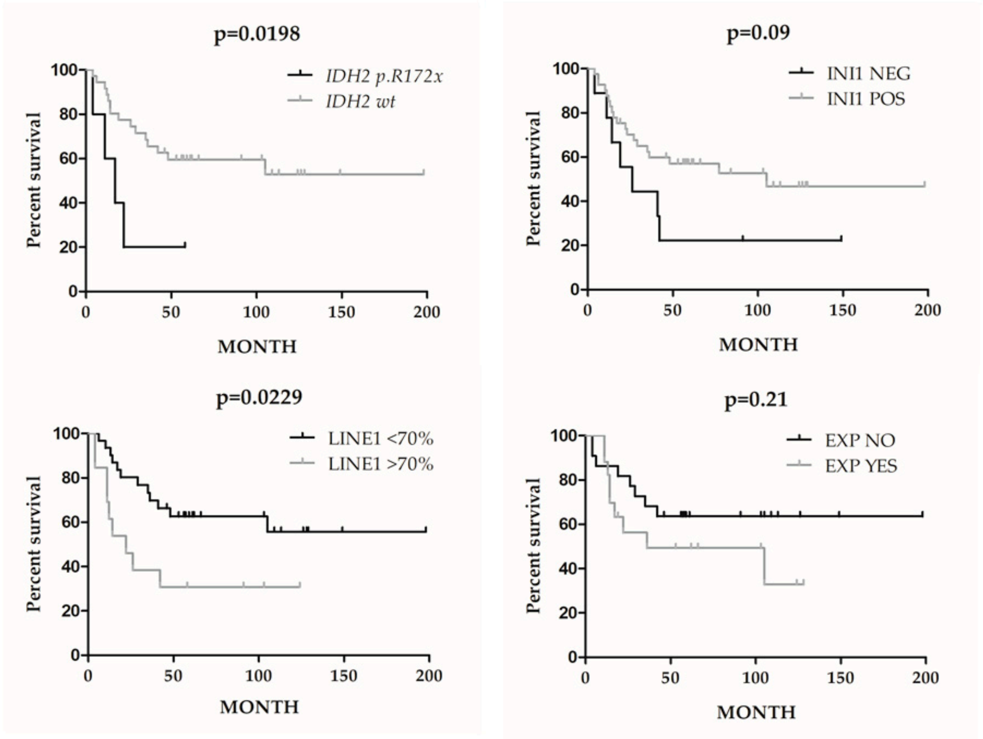

Figure S2. Univariable Survival Analyses according to INI1, IDH2, LINE-1 status and professional exposure.
